# Assessment tools for elder abuse: scoping review

**DOI:** 10.1590/1980-220X-REEUSP-2022-0115en

**Published:** 2022-12-05

**Authors:** Renata Clemente dos Santos-Rodrigues, Bárbara Maria Lopes da Silva Brandão, Gleicy Karine Nascimento de Araújo-Monteiro, Emanuella de Castro Marcolino, Ronei Marcos de Moraes, Rafaella Queiroga Souto

**Affiliations:** 1 Universidade Federal da Paraíba, Centro de Ciências da Saúde, Programa de Pós-Graduação em Enfermagem, João Pessoa, PB, Brazil.; 2 Centro Universitário Unifacisa, Departamento de Enfermagem, Campina Grande, PB, Brazil.; 3 Universidade Federal da Paraíba, Centro de Ciências Exatas e da Natureza, Departamento de Estatística. João Pessoa, PB, Brazil.

**Keywords:** Violence, Aged, Validation Studies, Elder Abuse, Forensic Nursing, Violência, Idoso, Estudo de Validação, Abuso do Idosos, Enfermagem Forense, Violencia, Anciano, Estudio de Validación, Abuso de Ancianos, Enfermería Forense

## Abstract

**Objective:**

to map assessment tools for elder abuse and determine the psychometric properties of each one.

**Method:**

scoping review developed according to recommendations of the JBI Institute Reviewer’s Manual in databases and gray literature.

**Results:**

seventeen tools were identified for measuring situations of elder abuse. They were categorized into 1) Tools for assessment of risk for abuse, and 2) Tools for identification of abuse. According to risk for abuse, Vulnerability to Abuse Screening Scale was the most prevalent in the literature, with factorial analysis acceptable through four domains, and good internal reliability (0,74). Therefore, Assessment Tool for Domestic Elder Abuse comprises the assessment of six types of elder abuse; however, the study shows psychometric limitation since the internal structure was not evaluated by validity evidences.

**Conclusion:**

seventeen tools to determine the occurrence or risk for elder abuse were identified with different psychometric properties. We recommend the use of more than one of the tools identified for an appropriate measurement of elder abuse situations given the complexity of the phenomenon and the lack of a single instrument that contemplates all its consequences and forms of expression.

## RESUMO

**Objetivo:**

mapear ferramentas de avaliação do abuso de idosos e determinar as propriedades psicométricas de cada uma.

**Método:**

revisão do escopo desenvolvido de acordo com recomendações do Manual do Revisor do Instituto JBI em bancos de dados e literatura cinzenta.

**Resultados:**

foram identificadas dezessete ferramentas para medir situações de maus-tratos a idosos. Elas foram categorizadas em 1) Ferramentas para avaliação de risco de abuso, e 2) Ferramentas para identificação de abuso. De acordo com o risco de abuso, a Escala de Vulnerabilidade ao Abuso foi a mais prevalente na literatura, com análise fatorial aceitável através de quatro domínios, e boa confiabilidade interna (0,74). Portanto, a Ferramenta de Avaliação para Abuso Doméstico de Idosos compreende a avaliação de seis tipos de abuso de idosos; entretanto, o estudo mostra limitação psicométrica, uma vez que a estrutura interna não foi avaliada por evidências de validade.

**Conclusão:**

dezessete ferramentas para determinar a ocorrência ou risco de abuso de idosos foram identificadas com diferentes propriedades psicométricas. Recomendamos o uso de mais de uma das ferramentas identificadas para uma medição apropriada das situações de abuso de idosos, dada a complexidade do fenômeno e a falta de um único instrumento que contemple todas as suas consequências e formas de expressão.

## RESUMEN

**Objetivo:**

mapear las herramientas de evaluación del maltrato a personas mayores y determinar las propiedades psicométricas de cada una de ellas.

**Método:**

revisión de alcance desarrollada según las recomendaciones del Manual del Revisor del Instituto JBI en bases de datos y literatura gris.

**Resultados:**

se identificaron diecisiete herramientas para medir situaciones de maltrato a personas mayores. Se clasificaron en 1) Herramientas para la evaluación del riesgo de maltrato, y 2) Herramientas para la identificación del maltrato. Según el riesgo de abuso, la Escala de Detección de Vulnerabilidad al Abuso fue la más prevalente en la literatura, con un análisis factorial aceptable a través de cuatro dominios, y una buena fiabilidad interna (0,74). Por lo tanto, el Instrumento de Evaluación del Abuso Domestico en Personas Mayores comprende la evaluación de seis tipos de maltrato a los ancianos; sin embargo, el estudio muestra limitación psicométrica ya que la estructura interna no fue evaluada por las evidencias de validez.

**Conclusión:**

e identificaron diecisiete herramientas para determinar la ocurrencia o el riesgo de maltrato a los ancianos con diferentes propiedades psicométricas. Se recomienda el uso de más de una de las herramientas identificadas para una adecuada medición de las situaciones de maltrato a los mayores dada la complejidad del fenómeno y la falta de un único instrumento que contemple todas sus consecuencias y formas de expresión.

## INTRODUCTION

The longevity observed in several countries reflects the improvement in the life of the population. However, there is concern with the quality of this aging process, since population aging also brought a growing number of vulnerable and dependent older adults of the civil society, state and family^(1)^. Elder abuse characterizes a public health problem with relevant consequences for their health^(2)^ and its incidence is related to sex, age, education, social support, depression, cognitive function, functional dependence for the performance of daily tasks and others^(3)^.

Elder abuse can be defined as “a single or repeated act, or lack of appropriate action occurring within any relationship where there is an expectation of trust which causes harm or distress to an older person”^(4)^. As this is a dynamic phenomenon, there is no consensus on the risk factors for its occurrence^(5)^, but some characteristics deserve attention because they are indicators of risk for abuse: female gender, advanced age, precarious physical and/or mental health, functional dependence, cognitive impairment, financial dependence, low income, conflicting intra-family relationship, social isolation, lack of social support and abuse of substances that can cause addiction or dependence^(3,5,6)^.

Recognizing the risk factors and suggestive signs of abuse is essential when tackling the phenomenon in search for the consolidation of appropriate public policies to face it^(3)^, and for that reason, it is essential to use appropriate instruments to support health professionals with the early detection of abuse. However, the tools used in clinical health practice must have valid and reliable psychometric characteristics that enable the measurement of a construct.

Measurement consists of the ability to measure whereas the construct is related to any abstract mental process sufficient to be objectively and directly quantified, and both vary according to their capacity of abstraction, complexity and stability^(7)^. In psychometry, a construct can also be called a latent trait, which in turn is a psychological and/or behavioral process that can be interpreted quantitatively according to the relationship between items of a scale or instrument^(8)^.

The reliability and validity of an instrument are represented by psychometric theories and techniques^(9)^. The validity of an instrument indicates the appropriateness of its measures to determine the studied behavior. In psychometry, the content, criteria and construct validation are the most common types of validation. Reliability consists of the test’s accuracy to predict the latent trait and is measured by reliability coefficients; the most commonly used is Cronbach’s alpha (〈), even though other statistical models can be adopted, for example, the Rulon coefficient (rtt), the Guttman-Flanagan and Kuder-Richardson (KR-20)^(8)^.

Considering the above, as well as the complexity of the phenomenon, the aim of the present study was to map the assessment tools for elder abuse and determine the psychometric properties of each one.

## METHOD

### Design of Study

The recommendations of the JBI Institute Reviewer’s Manual^(10)^ were followed in this literature scoping review, covering the steps: 1) Research strategy; 2) Source of screening and selection of evidence; 3) Data extraction and; 4) Analysis and presentation of results.

The guiding question was developed from the mnemonic PCC, in which P refers to participants (people aged 60 years or over), C to the concept (instruments for measuring the situation of abuse) and C to the context (elder abuse), namely “what are the validated tools to measure situations of elder abuse?”. This study was registered in the Open Science Framework (OSF) under protocol osf.io/nfq6m.

### Selection Criteria

The review included studies in all languages conducted with older adults (aged 60 years or over), using some tool to identify the situation of abuse, including validation, translation and cross-cultural adaptation studies. It excluded those tools mentioned in the literature that were not validated.

**Chart 1.**
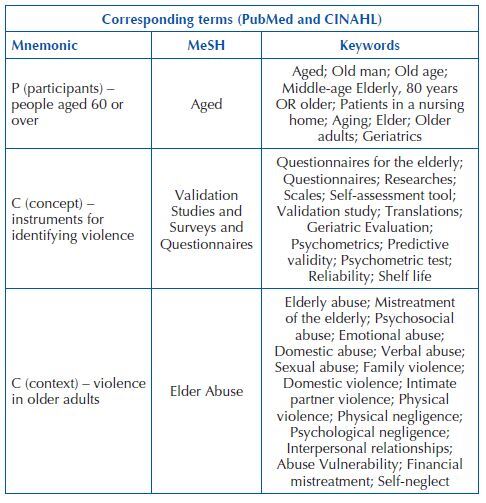
Selection of terms corresponding to the PCC mnemonic – João Pessoa, PB, Brazil, 2020.

The initial search was performed on MEDLINE (PubMed) and CINAHL (Cumulative Index to Nursing and Allied Health Literature) in order to find keywords corresponding to the mnemonic PCC using the following MeSH (Medical Subject Headings): Aged; Surveys and Questionnaires; and Elder Abuse, crossed with the Boolean operator AND, resulting in the strategy [(Aged) AND (Surveys and Questionnaires OR Validation Studies) AND (Elder Abuse)]. The terms found are shown in Chart 1.

The complete strategy generated was the following: [(“Aged” OR “Old man” OR “Old age” OR “Middle-age Elderly, 80 years or older” OR “Patients in a nursing home” OR “Aging” OR “Elder” “Older adults” OR “Geriatrics”) AND (“Questionnaires for the elderly” OR “Questionnaires” OR “Researches” OR “Scales” OR “Self-assessment tool” OR “Validation study” OR “Translations” OR “Geriatric Evaluation” OR “Psychometrics” OR “Predictive validity” OR “Psychometric test” OR “Reliability” OR “Shelf life”) AND (“Elderly abuse” OR “Mistreatment of the elderly” OR “Psychosocial abuse” OR “Emotional abuse” OR “Domestic abuse” OR “Verbal abuse” OR “Sexual abuse” OR “Family violence” OR “Domestic violence” OR “Intimate partner violence” OR “Physical violence” OR “Physical negligence” OR “Psychological negligence” OR “Interpersonal relationships” OR “Abuse Vulnerability” OR “Financial mistreatment” OR “Self-neglect”)]. The crossing was performed according to the specificity of each portal.

### Data Collection

The collection of documents was developed in the following databases and libraries: PubMed, CINAHL, Web of Science, Scopus, LILACS, Cochrane CENTRAL and PsychINFO. Gray literature was retrieved from the portals: CAPES Theses and Dissertations Portal, Academic Archive Online (DIVA), DART-Europe E-Theses Portal, Electronic Theses Online Service (EThOS) and Scientific Open Access Repository of Portugal (RCAAP). Flowchart 1 shows the selection of studies.

The documents on the portals were accessed remotely via the Federated Academic Community of the Coordination for the Improvement of Higher Education Personnel (CAPES) with login and password registered in the Integrated Management System for Academic Activities of the Universidade Federal da Paraíba.

The screening and selection of documents was performed between March and June 2020 by two trained reviewers; disagreements were discussed and clarified by a third reviewer.

### Data Analysis and Treatment

The information was extracted using an electronic spreadsheet containing the variables: author/year, title, database/library and identified instrument. The following variables were extracted from instruments: instrument, author/year, original language, country, if there was any cross-cultural adaptation, group collected, number of items, validation and reliability measures and indication of use.

## RESULTS

**Figure 1.**
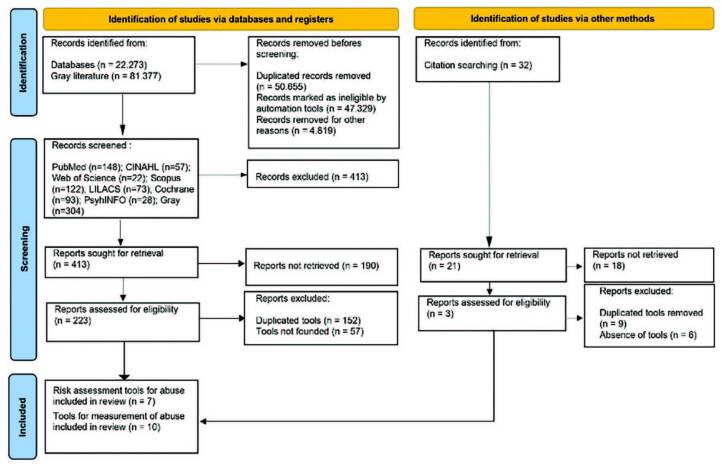
Flowchart of instrument selection adapted from PRISMA 2020.

The process of data search and selection is shown in Figure 1. The review followed the JBI recommendations for the adapted use of the Preferred Reporting Items for Systematic Reviews and Meta-Analyses (PRISMA – ScR).

**Chart 2.**
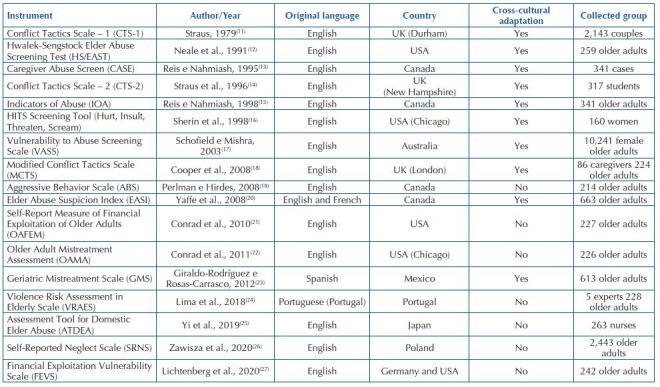
Instruments included in the scoping review, according to author, original language, country, cross-cultural adaptation and collected group – João Pessoa, PB, Brazil, 2020.

Seventeen screening tools for elder abuse were identified. Most were written in English, the cross-cultural adaptation and validation were performed with older adults, women, students and experts, as shown in Chart 2 (synthesis).

**Chart 3.**
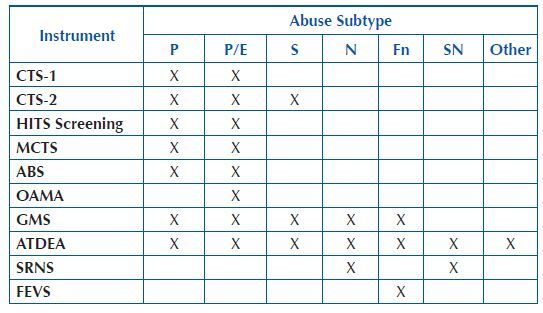
Instruments and subtypes of abuse measured – João Pessoa, PB, Brazil, 2020.

Chart 3 shows 10 tools to measure abuse identified in the literature, not only the risk for its occurrence. The tools were classified according to construct of abuse subtype as physical; psychological and emotional, or both; sexual; neglect; financial; and self-neglect.

P = physical abuse; P/E = psychological and emotional abuse; S = sexual; N = neglect; Fn = Financial; SN = self-neglect.

**Chart 4.**
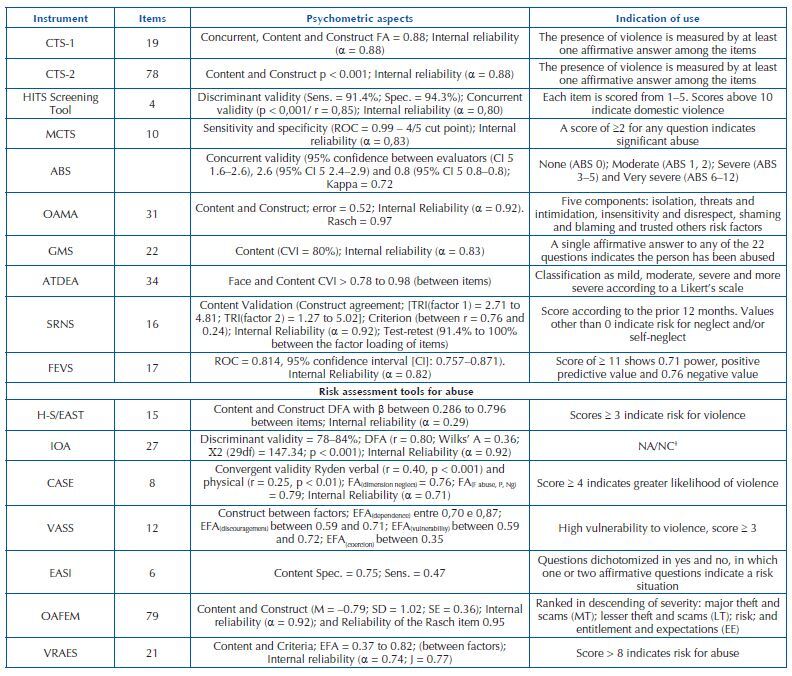
Description of abuse tools by number of items, psychometric aspects and indication of use – João Pessoa, PB, Brazil, 2020.

Chart 4 presents the descriptions of instruments by number of items, psychometric aspects and indication of use divided into two sections: tools for measuring abuse and tools for measuring the risk for abuse.

*FA = Factor Analysis; CFA = Confirmatory Factor Analysis; df = Degrees of Freedom; DFA = Discriminant Function Analysis; EFA = Exploratory Factor Analysis; Spe. = Specificity; Sens. = Sensitivity; ROC = Receiver Operating Characteristic Curve; SD = standard deviation; M = Average; r = correlation coefficient; ṡ^2^ = chi-square; J = Youden index; **〈 = Cronbach’s alpha; ^†^NA = Not applicable; NC = Not clarified.

## DISCUSSION

Elder abuse is a multifaceted problem with significant consequences for the health of older adults, becoming a public health problem^(2,28)^. It can present itself with physical, psychological and emotional abuse, sexual abuse, neglect, self-neglect and financial abuse^(28)^. The present review identified 10 instruments aimed at identifying multiple types of elder abuse, whether concomitantly or not.

The CTS-1^(11)^, CTS-2^(14)^, MCTS^(18)^, ABS^(19)^, HITS Screening^(29)^, GMS^(23)^, and ATDEA^(30)^ mediated the identification of physical and psychological elder abuse. Of these, it is important to discuss the set of instruments that make up the Conflict Tactics Scale from three perspectives: the CTS-1^(11)^, CTS-2^(14)^, and the MCTS^(18)^. All were applied in studies of groups of older adults, are part of a set of studies developed in the United States of America by the Family Research Laboratory and seek to identify situations of abuse within relationships, although only the MCTS was developed to diagnose abuse in older adults with dementia^(6)^.

In the original study, the use of MCTS indicated 27.9% of psychological abuse and 3.5% of physical abuse^(18)^. Similar data were found in the study developed by the same researchers with 32.7% of indicators of psychological abuse and 3.6% of physical violence^(6)^. In a more recent study conducted at the national level in Ireland^(31)^ with a sample of 2,311 subjects, one third of caregivers were involved with emotional abuse (35.9%) and 8% with physically abusive behavior.

All scales that determine physical abuse also presented a construct to identify psychological abuse, and this relationship between the two subtypes is justified, since episodes of psychological abuse commonly precede the occurrence of physical abuse, which is often less prevalent than the psychological in several contexts^(32,33,34,35,36)^.

Psychological abuse is the mental suffering that occurs as a result of verbal and non-verbal abuse^(37)^. The OAMA was the only scale in which the construct measures only psychological abuse self-reported by older adults.

The creators of OAMA adopted the Rasch model for analysis of the instrument^(22)^. The referred model is unidimensional and describes the representativeness of the degree of quality and property of a behavior and the relationship between the intended objects or events^(38)^. The model expresses a latent behavior or trait and is widely used in accordance with the Item Response Theory (IRT)^(39)^.

The SRNS was identified in the present review as having valid and reliable psychometric measures to measure neglect and self-neglect in older adults. Neglect characterizes a type of elder abuse and consists of the omission or denial of care to older adults by the other, whether a formal caregiver or not^(40)^. According to a study of 169 older adults developed in the state of Pernambuco, Brazil^(41)^, 58.5% of the population was in a situation of neglect. In a study conducted with 1,435 older adults in the city of Maharashtra, India, the estimated prevalence of neglect was 24.4%^(42)^. Self-neglect, in turn, consists of acts of threat against one’s own safety and health by refusing self-care^(40)^. Prevalence indicators vary according to the context and population. A study of 181 Chinese older adults living alone indicated a prevalence of 23.2%^(43)^, while in a longitudinal study in Chicago with 2,885 participants, the three-year indicator was estimated at 8.4%^(44)^.

One of the interfaces of elder abuse is financial exploitation. It can occur simultaneously with other forms of violence, expressing the need for adequate recognition by health professionals in order to guarantee protection for older adults^(45)^. The World Bank indicates the term “financial violence” as damage caused to the individual resulting from exploitation^(46)^. This type of violence is more prevalent in male older adults and perpetrated by an unknown person^(45)^. These characteristics are atypical when compared with other forms of violence against older adults, in which prevalence is higher in the female sex and the abuser is an intrafamily member.

FEVS was one of the instruments identified to determine the occurrence of financial abuse in older adults. The ROC curve represents the discriminatory power of the model that represents the study participants with regard to the studied outcome; the larger the area under the curve, the better its discriminatory power. Thus, models with an area less than or equal to 0.70 do not have discriminatory power, values between 0.70 and 0.80 are considered acceptable discriminatory power, and an area greater than 0.80 indicates excellent discriminatory power^(47)^.

The World Health Organization^(48)^ defines sexual violence as “any sexual act, attempt to obtain a sexual act directed against a person’s sexuality using coercion, by any person regardless of their relationship to the victim, in any setting, including but not limited to home and work”. This type of violence is still underreported, and the lack of recognition of cases of sexual violence makes the older adult vulnerable and with low support from effective health policies related to the theme.

The HITS instrument^(29)^ was identified in two studies, even though it was not directed to a population of older adults^(49,50)^. In the literature, a variation of the HITS (extended version) was found; the E-HITS (Extended – Hurt, Insult, Threaten, Scream), which involves the facet of sexual violence, although its application in older adults has not been identified.

The HITS cross-cultural adaption for Brazil was performed for application to older adults according to the steps^(51)^, including initial translation, synthesis of translations, back-translation, expert committee and pre-test. The authors of the cross-cultural adaptation indicated good reliability through the analysis of internal reliability and the intraclass correlation coefficient^(50)^.

The scope of the GMS^(23)^ and the ATDEA^(30)^ is to identify five types of abuse specifically in older adults, namely physical, psychological, sexual, neglect and financial.

The GMS was developed in three steps: document review, contextualization and scale development. The tool is composed of dichotomous questions (yes or no), underwent content validation (by experts and older adults) and construct validation (factor analysis). The instrument reliability was considered high and determined by the internal reliability coefficient. There were variations between the dimensions: 〈 = 0.82 for psychological abuse; 〈€= 0.72 physical abuse; 〈 = 0.55 financial abuse; 〈 = 0.80 for neglect; and 〈 = 0.87 for sexual violence^(23)^. Although reliability for the financial dimension was insufficient, the instrument was considered reliable because of its appropriate general coefficient (〈 = 0.83).

Construct and reliability validations were not performed for the ATDEA, but two rounds of tool assessment were carried out with nurses who provide home care and had confirmed experience with the theme of elder abuse. In the first round, 56 nurses discussed the 38 items and face validity of the instrument, nine researchers compiled the information into categories by consensus and only 36 items went to the second round. In this phase, the content validation of the instrument was performed with 207 nurses and it was categorized by subtypes of violence^(30)^.

The VASS was planned within the scope of a longitudinal project containing three temporal cuts, and had a final sample of 10,421 female older adults from Australia. The instrument contains 12 items, of which ten were extracted from the H-S/EAST and two questions were added to the instrument screening^(17)^. Older adults with a score greater than or equal to three are classified as being at risk for violence. The approximation of items and interpretation between the two scales explains the use of VASS in 15 studies, and in six of them, the concomitant use of the VASS and H-S/EAST was indicated.

The original version showed validity and reliability, the construct was defined by EFA through four domains: vulnerability, discouragement, dependence and coercion, the latter with low reliability compared to the other three^(17)^. The cross-cultural adaptation to Turkish language was performed with 140 older adults presenting good internal reliability (〈€= 0.819) in data analysis. Researchers used the Geriatric Depression Scale for the criterion validity test, in which was achieved moderate correlation (r = 0.57) between scores of the scales^(52)^.

The Brazilian version of the instrument discussed above was developed in two dissertations published in 2014 on the Brazilian portal of the Coordination for the Improvement of Higher Education Personnel (Portuguese acronym: CAPES)^(53,54)^. The version developed in Belo Horizonte included 151 older adults and presented equivalence with the original instrument between the dimensions of validity, as well as good internal reliability (KR-20 = 0.69) and excellent reproducibility (Kp = 0.92)^(53)^. The version developed in the state of Rio Grande do Norte reached similar findings (KR-20 = 0.68)^(55)^.

Although the two scales discussed so far (VASS and H-S/EAST) are indicated as screening tools for identifying the risk for abuse, in some studies, they were applied concomitantly as a conclusive measure of the occurrence of violence. For example, in a study developed in Singapore with 400 older adults, the conclusion was that 8.3% were victims of some form of violence^(56)^.

Although the BASE was not included among the sample articles, it was applied together with the IOA and CASE in an intervention study of older adults with the aim to assess screening aspects for physical, financial and psychological abuse^(13)^. Both instruments were incorporated into the Project Care, applied by a multidisciplinary team to propose interventions involving three main tenets for its execution: tools, professionals and elements, in search for the empowerment of older adults in situations of abuse.

The IOA, was applied prospectively to 341 older adults and presented discriminant validation related to the time between cases of abuse (84.4%) and non-abusive cases (99.2%). The IOA was adapted for the Spanish language with 231 older adults and the results indicated high internal reliability between items by Cronbach’s alpha (〈€= 0.98) and it indicated sensitivity = 0.94 and specificity = 0.85 for the score of 16^(57)^.

The CASE was designed with dichotomous questions (yes and no) in search for evidence of abusive caregivers from a physical, psychosocial, material, neglect or financial perspective. The aim of the eight items of the instrument is to understand the caregiver’s behavior, such as: “Do you sometimes have problems controlling your temper or aggression?”^(13)^. Cross-cultural adaptations for Brazilian Portuguese, Italian, Persian, Turkish, and Iranian languages were identified^35,58,59,60^.

Identifying the potential perpetrator of violence is critically important to combat the phenomenon under discussion. Among the instruments identified, only the CASE offers this scope. In a study conducted in Spain with 72 primary care teams, was identified a 33.4% prevalence of risk for abuse. In the logistic regression analysis, the risk for abuse was 2.75 times higher among overworked caregivers, 2.06 higher among anxious caregivers and 4.66 higher among those with weak interpersonal relationships, while those with aggressive behavior had a 7.24 higher probability of showing abusive behavior^(61)^. A study developed in Brazil showed 30% of indicators of abuse among caregivers, with higher chances among those who consume alcohol and among depressed caregivers^(62)^. These indicators demonstrate the need for supportive policies and interventions in the family environment.

In 2008, an EASI validation study conducted with 663 older adults from Canada and the USA was published with the aim to measure the suspicion of abuse against older adults in areas of physical violence and neglect. The instrument was validated by doctors, nurses and social workers and the results from data collection were compared with a blind assessment of social work (Social Work Evaluation – SWE)^(20)^.

During the approach to the victim of violence, the professional must develop the sensitivity of perceiving signs of the different types of violence. Considering neglect and/or abandonment by the patient’s body presentation, observing behavioral signs such as facial expressions of apathy, astonishment or anguish in the presence or absence of the immediate caregiver, in addition to agreement or not in relation to the information reported by the older adult^(63)^. Although the behavioral dimension is fundamental for screening, only the EASI tool includes it in its risk scope. The item is written as follows: “Elder abuse may be associated with findings such as poor eye contact, withdrawn nature, malnourishment, hygiene issues, cuts, bruises, inappropriate clothing or medication compliance issues. Have you noticed any of these today or in the last 12 months?”^(20)^.

The VRAES was developed with 228 older adults in Portugal^(24)^. The authors of the instrument used the Youden index (J) to estimate the specificity and sensitivity of results. The Youden indicator measures the distance from the ROC curve (maximum vertical distance) and the chance line (diagonal) by optimizing the difference in biomarkers and then, the specificity and sensitivity, thus, it is essential for diagnostic accuracy^(64)^.

The most recent screening scale identified in the present study was the OAFEM, aimed at identifying the financial exploitation of older adults. The Rasch model was used for validation while internal consistency was used for internal reliability. Despite being a measuring instrument for the occurrence of violence, it was discussed in the present study because of its one-dimensional characteristic. Items 79, 54 and 30 were ranked in descending severity in four groups: major theft and scams (MT); lesser theft and scams (LT); risk for financial abuse; and entitlements and expectations (EE)^(21)^.

Finally, it is noteworthy that in the O AFEM was used the mathematical theoretical model of Item Response Theory (IRT), in which the intention in using an instrument is applied to the item’s ability to influence the proposed outcome, in the search for reduction of items, while in the Classical Test Theory (CTT), the total score of the instrument is interpreted to measure the outcome by adding all items^(8)^.

The limitation of this study includes the concepts related to standardization the construction of instruments. Besides the great number of instruments, we observe low psychometric efficiency to determine the phenomenon.

## CONCLUSION

Although a significant number of instruments available to measure elder abuse has been identified, all have singularities and strengths for making a situational diagnosis of abuse. However, none of them covered the assessment from the professional’s point of view regarding the convergent or divergent behavior in relation to the older adult’s report at the time of data collection.

The verbal indication of issues related to violence by the older adults is essential to identify the phenomenon or its risk. However, we recommend to include questions related to the professional and/or researcher’s judgement together with the patient’s and/or participant’s report. This is relevant because it takes into account the observations of unreported signs and symptoms that are commonly omitted because of the older adult’s lack of understanding of violence and its consequences or fear of the perpetrator.

Seven instruments that aim to measure the risk for elder abuse were identified. The oldest and more widespread is the H-S/EAST, although its original version presented low accuracy. The VASS was developed based on the H-S/EAST although with better reliability indicators. The CASE and IOA were instruments developed in a three-year intervention research with good discriminant validity of risk for violence. The original EASI study did not indicate that the instrument reliability (assessment) was performed and had low sensitivity and moderate specificity. Although the validation and reliability steps have been performed for the OAFEM, as this is an extensive and specific instrument for financial violence, caution is required when using it to measure the risk for abuse.

Although there are several valid instruments for screening violence, all have unique characteristics with strengths and weaknesses, thus the recommendation to use more than one in the search for achieving a better situational risk diagnosis.
